# SARS-CoV-2 Infection Triggers Phosphorylation: Potential Target for Anti-COVID-19 Therapeutics

**DOI:** 10.3389/fimmu.2022.829474

**Published:** 2022-02-17

**Authors:** Bhaswati Chatterjee, Suman S. Thakur

**Affiliations:** ^1^ Chemical Science, National Institute of Pharmaceutical Education and Research, Hyderabad, India; ^2^ Proteomics and Cell Signaling, Centre for Cellular and Molecular Biology, Hyderabad, India

**Keywords:** COVID-19, SARS-CoV-2, phosphorylation, function, therapeutics

## Abstract

The SARS-CoV-2 infection triggers host kinases and is responsible for heavy phosphorylation in the host and also in the virus. Notably, phosphorylations in virus were achieved using the host enzyme for its better survival and further mutations. We have attempted to study and understand the changes that happened in phosphorylation during and post SARS-CoV-2 infection. There were about 70 phosphorylation sites detected in SARS-CoV-2 viral proteins including N, M, S, 3a, and 9b. Furthermore, more than 15,000 host phosphorylation sites were observed in SARS-CoV-2-infected cells. SARS-CoV-2 affects several kinases including CMGC, CK2, CDK, PKC, PIKFYVE, and EIF2AK2. Furthermore, SARS-CoV-2 regulates various signaling pathways including MAPK, GFR signaling, TGF-β, autophagy, and AKT. These elevated kinases and signaling pathways can be potential therapeutic targets for anti-COVID-19 drug discovery. Specific inhibitors of these kinases and interconnected signaling proteins have great potential to cure COVID-19 patients and slow down the ongoing COVID-19 pandemic.

## 1 Introduction

**Figure 1:**
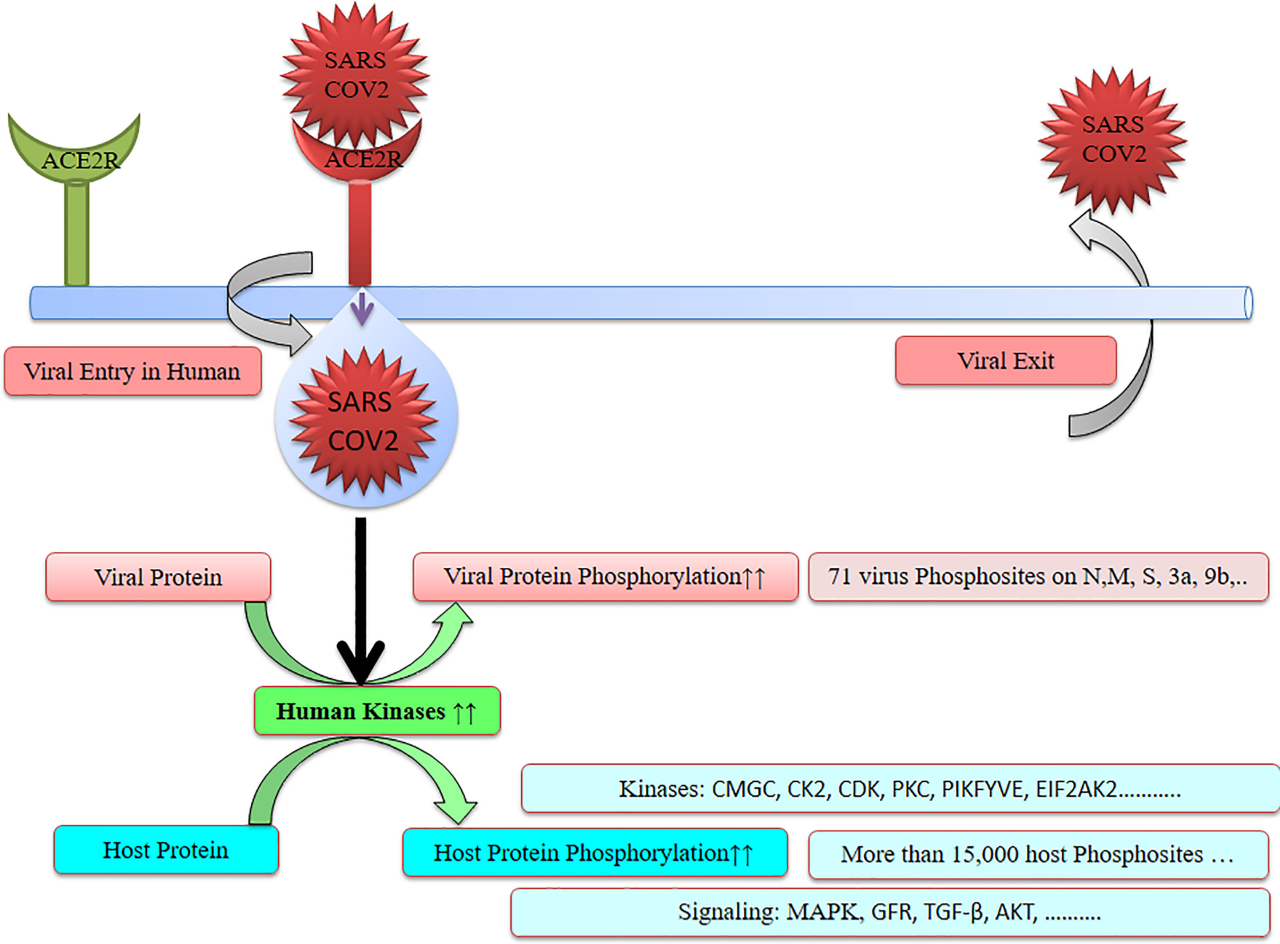
SARS-CoV-2 causes heavy phosphorylation.

**Figure 2:**
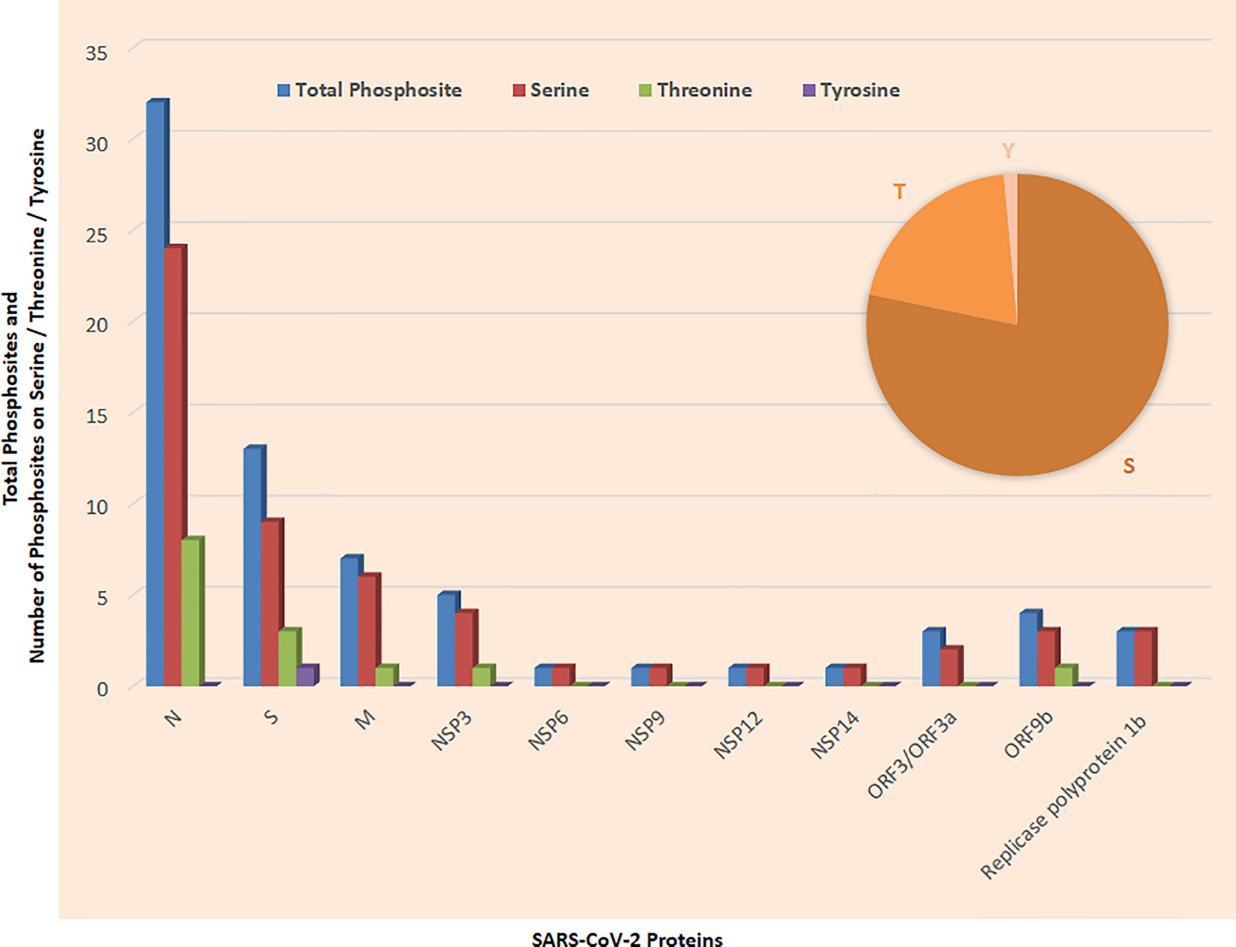
Phosphosites detected in SARS-CoV-2 proteins; inset shows phosphosites on Serine (S), Threonine (T), and Tyrosine (Y).

**Figure 3:**
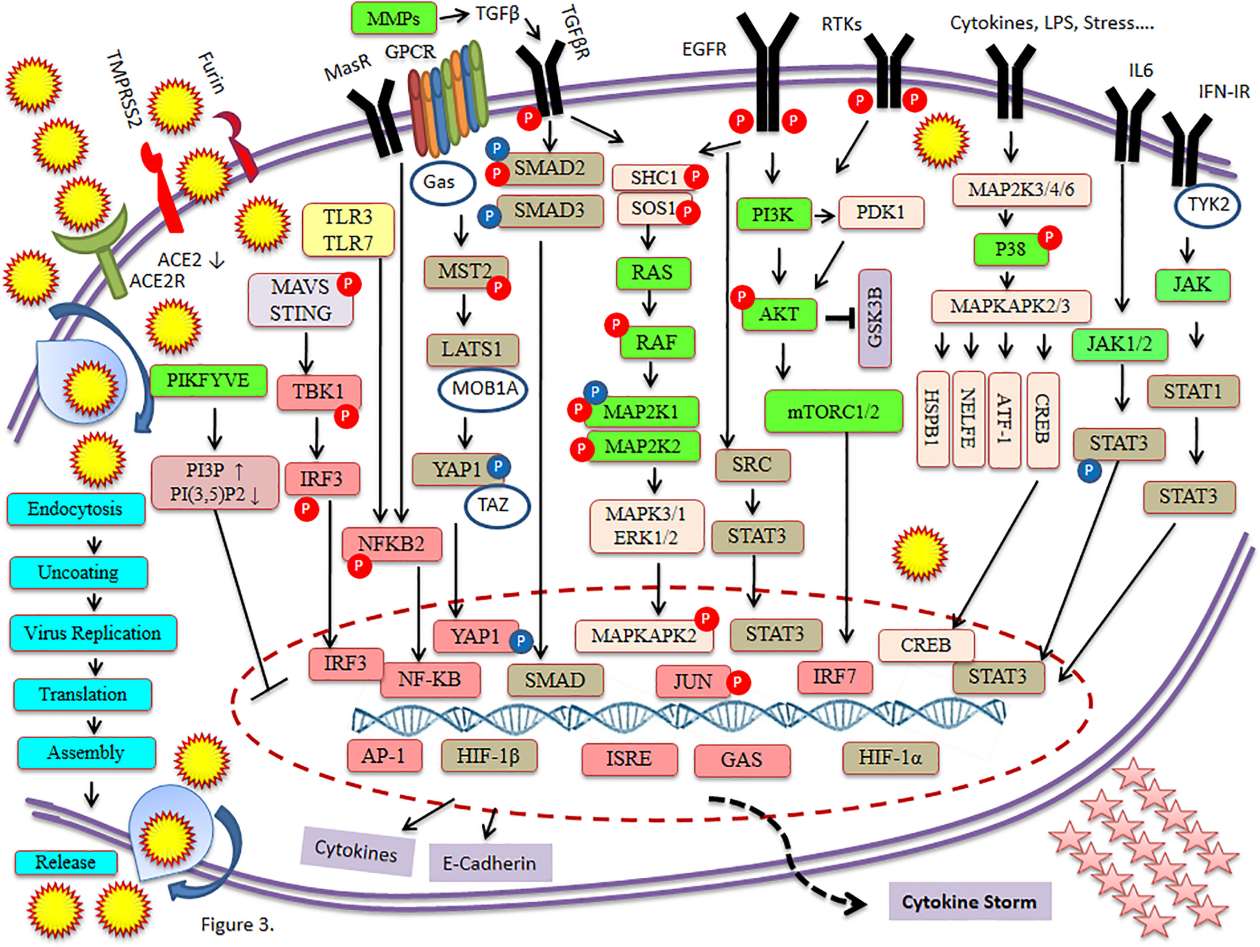
Major signaling pathways involved in COVID-19.

**Figure 4:**
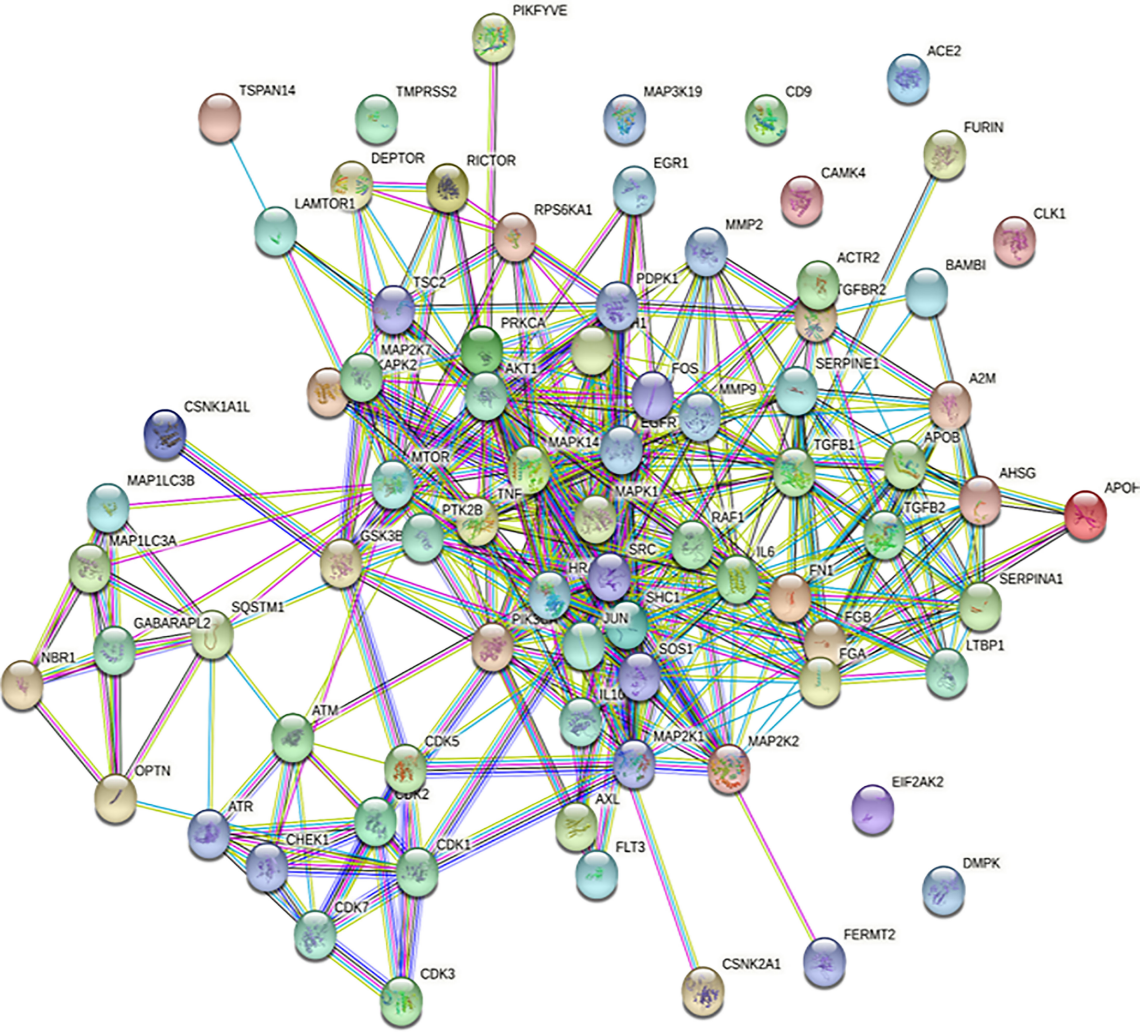
Interaction network of major proteins involved in COVID-19 at high confidence scores.

**Figure 5:**
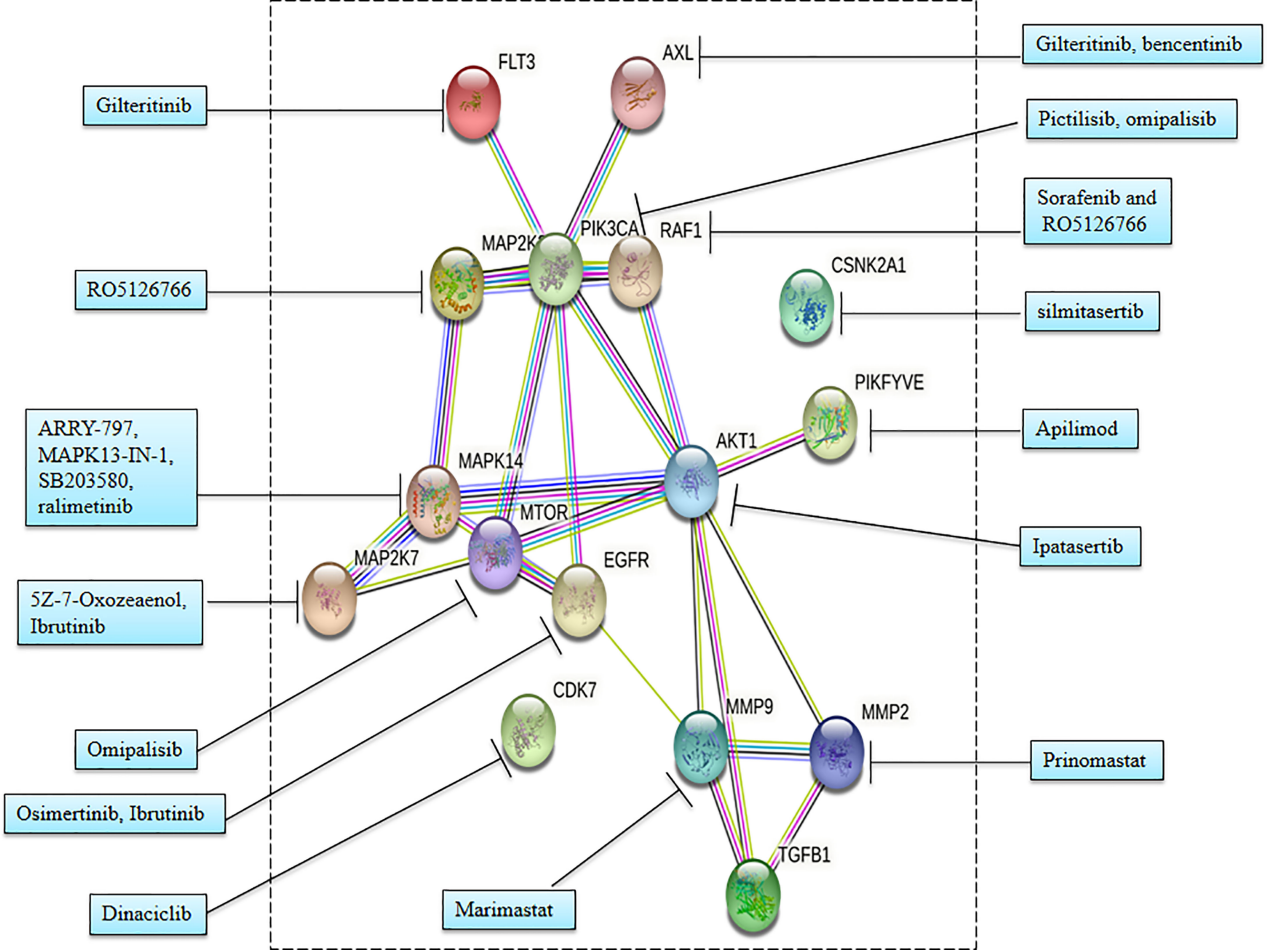
Interaction network of therapeutic targets for COVID-19 at high confidence scores.

SARS-CoV-2 has wreaked havoc globally and is responsible for billions of infections and millions of deaths during the ongoing COVID-19 pandemic. Further frequent appearances of new variants do not contribute to the slowing down of COVID-19. SARS-CoV-2 has also wreaked havoc inside the cell and made several changes including post-translational modification such as phosphorylation in the host (**Figure 1**). SARS-CoV-2 infection activates host kinase and causes heavy phosphorylation in the host (**Figure 1**) and also in the virus proteins (**Figure 2**). Furthermore, various host signaling pathways were activated due to SARS-CoV-2 infection (**Figure 3**). Notably, these differential phosphoproteins and interacting proteins are nicely interconnected (**Figure 4**). Some of these kinases and differential proteins are important targets for COVID-19 therapeutics (**Figure 5**). Several groups have attempted to study the global phosphorylation changes in SARS-CoV-2-infected cell lines using highly sensitive chimpanzee Vero E6, human colon cancer CaCo2, human lung cancer A549-ACE2, and induced pluripotent stem cell-derived AT2s (iAT2s) (**Supplementary Figures 1A–C**) (1–5).

SARS-CoV-2 infects alveolar epithelial type 2 cells (AT2s) that cause injury to the lungs and cause impaired gas exchange, while the mechanism and pathogenesis are still not known. Angiotensin-converting enzyme 2 (ACE2) and transmembrane serine protease 2 (TMPRSS2) play an important role in the entry of SARS-CoV-2 in humans (6). SARS-CoV-2 significantly infects AT2s that express both ACE2 and TMPRSS2 (7, 8). Furthermore, SARS-CoV-2 causes injury of AT2s that leads to severity and death of COVID-19 patients. Notably, AT2s function as progenitors of lung alveoli and help in the regeneration of injured epithelium and reduce the surface tension by secreting pulmonary surfactant stored in lamellar bodies (3).

SARS-CoV-2 genome encodes about 27 proteins, namely, 4 structural, 15 nonstructural, and 8 auxiliary proteins, and modulates host response by interaction with the host factor. Spike (S), envelope (E), membrane (M), and nucleocapsid (N) belong to the structural proteins (3, 5, 9, 10). Major viral proteins were significantly increased while the majority of host proteins were decreased during SARS-CoV-2 infection. Post-translational modification including phosphorylation occurs in the majority of SARS-CoV-2 virus proteins (**Supplementary Figures 1B, C**). SARS-CoV-2 infection is responsible for heavy phosphorylation in host system proteins and in viral proteins. Furthermore, it regulates several host signaling pathways (**Figures 1**, **3**). SARS-CoV-2 hijacked respiratory-specific processes of the host system and remodels several pathways including RNA processing, translation, and protein trafficking that leads to cell cycle stress (3). Phospho-proteomics studies have helped to understand the signaling pathways, host–pathogen interaction, pathophysiology of SARS-CoV-2 infection, and their consequences in humans (5).

Notably, identification of phosphorylation sites suggests potential therapeutic targets that help to find suitable kinase inhibitors against the virus (**Figures 1**, **3**, **5**). Identification of transcripts was achieved by transcriptomics and expressions of transcripts were confirmed by mass spectrometry-based proteomics. Davidson et al. have reported SARS-CoV-2 transcriptome using RNA sequencing along with proteome and phosphoproteome using tandem mass spectrometry. SARS-CoV-2 transcripts were similar to the expected coronavirus model (4). In another study, Stukalov et al. have infected a lung cancer cell line A549 expressing angiotensin-converting enzyme 2 (A549-ACE2 cells) with SARS-CoV-2 and studied the interaction of the virus with other proteins (interactome) and effectome including the impact of the virus on host proteome, transcriptome, phosphoproteome, and ubiquitome (5).

## 2 Phosphorylation Studies of SARS-CoV-2 Proteins

Mass spectrometry-based phospho-proteomics studies of SARS-CoV-2-infected Vero E6 cells identified 25 phosphosites (**Supplementary Figure 1**). Notably, the Vero E6 cell line has a very high susceptibility for SARS-CoV-2 infection and originated from the kidney of a female African green monkey. Furthermore, 21 phosphosites were detected on serine, 4 phosphosites were detected on threonine, whereas no phosphosites were detected on tyrosine. Serine phosphosites were detected on viral proteins such as M, N, nsp3, nsp14, and orf9b. Threonine phosphosites were detected on N (nc) viral proteins. These phosphosites belong to several kinase groups including CK2, GRK, ACTR2-ACTR2B-TGFbR2, CLK, p38, CDK2-CDK3-CDK1-CDK5, DMPK, RCK, ATM-ATR, PKB, CK1, and TLK (1). The phosphosites S213, S212, S1826, and S214 belong to Casein kinase 2 (CK2); T76 belongs to G protein-coupled receptor kinases (GRK) and S79 belongs to the p38 group (1).

Davidson et al. reported 44 phosphosites (**Supplementary Figure 1**) and 500 viral peptides identified in SARS-CoV-2-infected cells that cover most of the encoded protein by SARS-CoV-2 genome with the observation of a 24-nt in-frame deletion in more than half of subgenomic mRNA that encodes the spike (S) glycoprotein (4). Furthermore, Klann et al. (2020) reported that 33 modification sites were present on 6 viral proteins while their function and understanding of regulation are still unknown (2). Hekman et al. found 8 SARS-CoV-2 proteins along with 2 viral phosphoproteins (**Figure 3**). Furthermore, Hekman et al. reported that host human enzyme causes phosphorylation of SARS-CoV-2 proteins including M and N proteins. There were 9 phosphosites detected on N proteins including the linker region between the receptor-binding domain (RBD) and dimerization domains. Additionally, phosphosites were also detected in the C-terminal cytoplasmic domain (3). Stukalov et al. reported 23 phosphorylation sites on 5 phosphoproteins of SARS-CoV-2 (5).

Overlapping of several phosphosites was reported in various studies. Notably, phosphorylation of S213 in M protein was detected in all 5 studies (1,2,^3^–^5^) (**Table 1**). Interestingly, phosphorylation of S23, S26, S79, S176, T198, S201, S202, T205, and S206 of N protein, and S214 of M protein was detected in 4 studies (**Table 1**). Furthermore, phosphorylation of T24, T141, S180, S183, and S184 in N protein; S212 in M protein; S1826 in NSP3; and S50 in ORF9b was detected in 3 different studies (**Table 1**). Furthermore, phosphorylation of T76, S78, S105, T166, S194, S197, and S412 in N protein; S173 and S211 in M protein; T791 in S protein; and S53 and T72 in ORF9b was detected in two studies (**Table 1**).

**Table 1:** Phosphorylation sites identified in SARS-CoV-2 proteins.

Sl. No.	Protein Name	Phosphosites location on			References
		Serine	Threonine	Tyrosine	
1	N	S2			(4)
2	N	S21			(5)
3	N	S23			(1–4)
4	N		T24		(1, 4, 5)
5	N	S26			(1–3, 5)
6	N	S33			(2)
7	N		T76		(1, 4)
8	N	S78			(2, 4)
9	N	S79			(1–4)
10	N	S105			(1, 4)
11	N		T141		(2, 4, 5)
12	N		T166		(4, 5)
13	N	S176			(1, 2, 4, 5)
14	N	S180			(2, 4, 5)
15	N	S183			(1, 2, 4)
16	N	S184			(1, 2, 4)
17	N	S186			(2)
18	N	S188			(2)
19	N	S190			(2)
20	N	S194			(1, 4)
21	N	S197			(1, 3)
22	N		T198		(1–4)
23	N	S201			(1, 2, 4, 5)
24	N	S202			(1, 2, 4, 5)
25	N		T205		(1–4)
26	N	S206			(1–4)
27	N	S310			(5)
28	N		T391		(4)
29	N	S410			(5)
30	N	S412			(3, 5)
31	N	S413			(2)
32	N		T417		(2)
33	M	S172			(2)
34	M	S173			(2, 5)
35	M		T208		(4)
36	M	S211			(1, 4)
37	M	S212			(1, 4, 5)
38	M	S213			(1–5)
39	M	S214			(1, 3–5)
40	S		T29		(4)
41	S	S31			(4)
42	S		T240		(4)
43	S	S349			(4)
44	S	S459			(4)
45	S	S637			(4)
46	S	S640			(4)
47	S			Y789	(4)
48	S		T791		(4, 5)
49	S	S816			(4)
50	S	S1161			(4)
51	S	S1196			(4)
52	S	S1261			(4)
53	NSP3		T504		(4)
54	NSP3	S660			(4)
55	NSP3	S661,			(4)
56	NSP3	S794,			(4)
57	NSP3	S1826			(1, 4, 5)
58	Nsp6	S50			(2)
59	NSP9	S5			(4)
60	NSP14	S56			(1)
61	ORF 3a	S248			(2)
62	ORF 3a	S252			(2)
63	ORF9b	S50			(1, 2, 5)
64	ORF9b	S53			(1, 5)
65	ORF9b	S63			(5)
66	ORF9b		T72		(2, 5)
67	Replicase polyprotein 1b	S723			(2)
68	Replicase polyprotein 1b	S2644			(2)
69	Replicase polyprotein 1b	S5981			(2)
70	NSP12	Probably S, Site is not known Peptide sequence: GFFKEG**SS**VELK			(4)
71	ORF 3a	Probably T or S, Location site is not known Peptide sequence: QGEIKDA**T**P**S**DFVR			(4)

The discrepancy in the detection of phosphosites in various studies may be due to various parameters including a selection of different cell lines, the efficiency of SARS-CoV-2 isolates, sample preparation, enrichment method, instrumental condition, and chromatography condition (**Table 2**). The cell lines used for the studies include Vero E6 from African green monkey kidney epithelial (1, 4), colon epithelial cell line Caco-2 (2), iPSC-derived alveolar epithelial type 2 cells (iAT2s) (3), and A549-ACE2 cells from human (5). The source of SARS-CoV-2 was different in each study such as SARS-CoV-2 isolate BetaCoV/France/IDF0372/2020, SARS-CoV-2 that was isolated from samples of travelers returning from Wuhan (China) to Frankfurt (Germany), SARS-CoV-2 stocks (isolate USA_WA1/2020), SARS-CoV-2 strain England/2/2020 (VE6-T), and SARS-CoV-2-MUC-IMB-1. Furthermore, different types of high-resolution mass spectrometers were used in the studies such as Mass Spectrometer Orbitrap Exploris 480 (1, 5), Orbitrap Fusion Lumos (2, 4), and Q-Exactive HF-X (3). Chromatography conditions were different in each study including gradient length 70 min, 120 min, 135 min, 140 min, and 150 min using the nano-LC system. Phosphopeptide enrichment was done using TiO_2_ and FeNTA. Data acquisition was done in different modes: either Data-dependent analysis (DDA) or Data-independent analysis (DIA), or both DDA and DIA in these studies. Different spray voltages were used to ionize the phosphopeptides in each study such as 2,000 V, 2,100 V, 2,200 V, 2,600V, and 2,650 V (**Table 2**) while 275°C ion transfer tube/capillary temperature were used in 3 studies, and 250°C and 300°C were also used in other studies. Notably, full scan range MS spectra were different in each study such as 350–1400 m/z, 350–1500 m/z, 375–1550 m/z, and 300–1400 m/z at a resolution of 120,000 at 200 m/z, while in one study, full scan range MS spectra was 400–1000 m/z at a resolution of 60,000 at 200 m/z. Notably, fragmentation of selected peptide occurred in HCD using different normalized collision energy such as NCE 29%, NCE 38%, and NCE stepped 25–27.5–30% in each study while NCE 30% was used in two studies (**Table 2**).

**Table 2:** Detail of experimental settings of phosphorylation studies.

Sl. No	Experimental Settings	Phospho Studies				
		Bouhaddou et al., 2020 (1)	Klann et al., 2020 (2)	Hekman et al., 2020 (3)	Davidson et al., 2020 (4)	Stukalov et al., 2020 (5)
1	Cell line	Vero E6 (African green monkey kidney epithelial)	Colon epithelial cell line Caco-2, (Human)	iPSC-derived alveolar epithelial type 2 cells (iAT2s) (Human)	Vero E6 (African green monkey kidney epithelial)	A549-ACE2 cells (Human)
2	Virus	The SARS-CoV-2 isolate BetaCoV/France/IDF0372/2020	SARS-CoV-2 was isolated from samples of travelers returning from Wuhan (China) to Frankfurt (Germany)	SARS-CoV-2 stocks (isolate USA_WA1/2020)	SARS-CoV-2 strain England/2/2020 (VE6-T)	SARS-CoV-2-MUC-IMB-1
3	Mass spectrometer	Orbitrap Exploris 480 (Thermo Fisher Scientific)	Orbitrap Fusion Lumos (Thermo Fisher Scientific)	Q-Exactive HF-X (Thermo Fisher Scientific)	Orbitrap Fusion Lumos (Thermo Fisher Scientific)	Orbitrap Exploris 480, (Thermo Fisher Scientific)
4	HPLC	Easy nLC 1200 (Thermo Fisher Scientific)	Easy nLC 1200 (Thermo Fisher Scientific)	Easy nanoLC1200 (Thermo Fisher Scientific)	nano-LC MSMS U3000-Proflow (Thermo Fisher Scientific)	EASY-nLC 1200 (Thermo Fisher Scientific)
5	Column	25 cm × 75 μm ID packed with ReproSil-Pur 1.9-μm particles	32 cm × 75 μm ID, packed with 1.9-μm C18 particles	EASY-Spray column, (ES803A, Thermo Scientific), 50 cm × 75 µm ID, PepMap RSLC C18, 2 µm	250 mm × 75 μm Acclaim PepMap C18 reverse-phase analytical column (Thermo Scientific)	50 cm × 75 μm ID packed with ReproSil-Pur C18-AQ 1.9-μm resin
6	Gradient	140 min	135 min	120 min	150 min	70 min
7	Phospho-peptide, enrichment	Yes, With Superflow bead slurry (QIAGEN)	Yes, With Fe-NTA Phosphopeptide enrichment kit (Thermo Fisher)	Yes, With Fe-NTA magnetic beads (CubeBiotech)	Yes, With TiO_2_-based and FeNTA phosphopeptide enrichment (Pierce)	Yes, With Fe3+-NTA AssayMAP cartridges/Bravo robot (Agilent)
8	Acquisition mode	Data-dependent analysis (DDA) and Data-independent analysis (DIA)	DDA	DDA	DDA	DIA
9	Spray voltage	2,000 V	2,600 V	2,100 V	2,200 V	2,650 V
10	Ion transfer tube/capillary temperature C	275°C	300°C	275°C	250°C	275°C
11	Full scan range MS spectra and resolution at 200 m/z	400–1,000 m/z at resolution of 60,000	350–1,400 m/z at resolution of 120,000	350–1,500 m/z at resolution of 120,000	375–1,550 m/z at resolution of 120,000	300–1,400 m/z at resolution of 120,000
12	Fragmentation	HCD (NCE 30%)	HCD (NCE 38%)	HCD (NCE 29%)	HCD (NCE 30%)	HCD (NCE stepped 25–27.5–30%)

## 3 Importance of Phosphorylation in Virus Proteins

**Figure 6:**
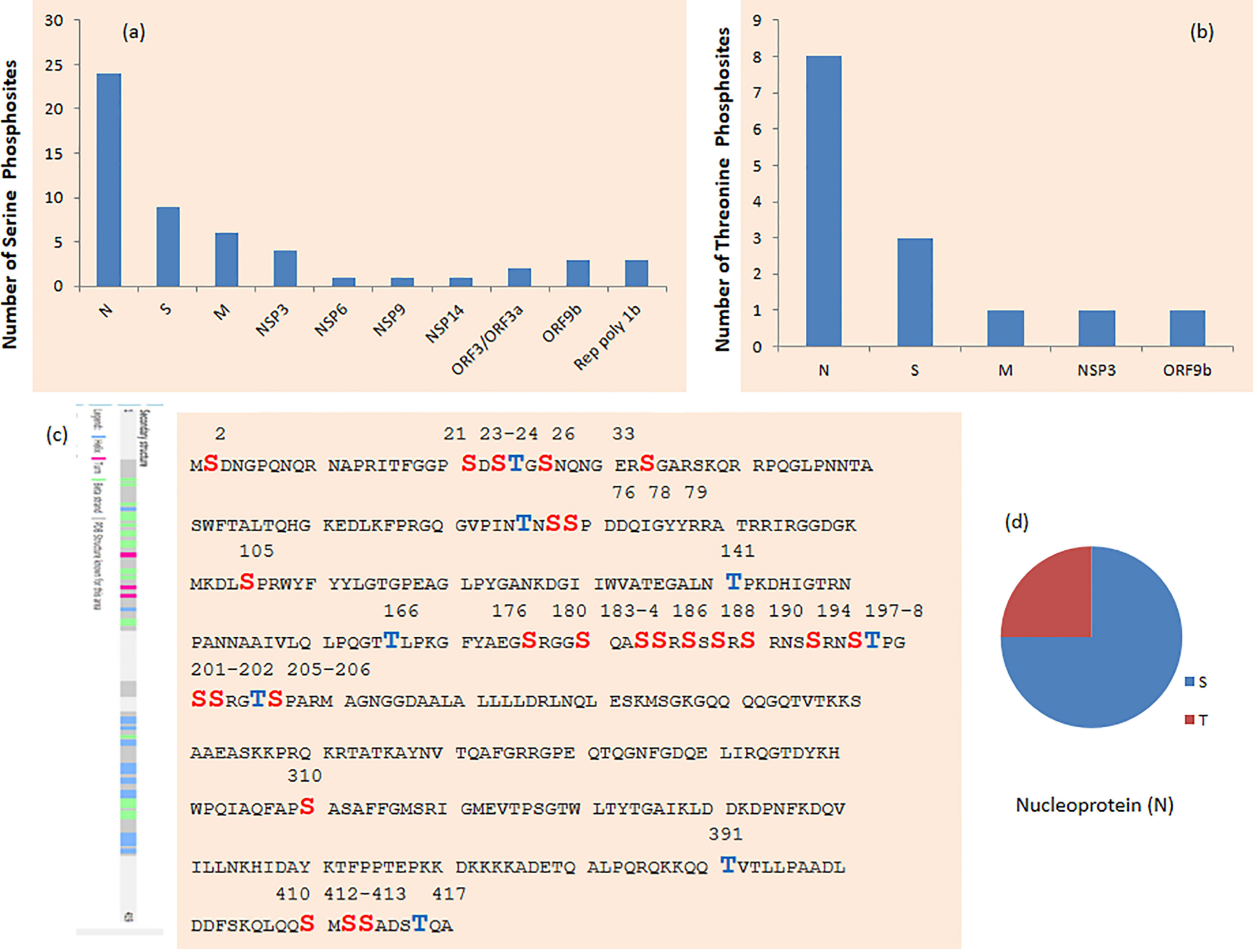
**(A)** Serine and **(B)** threonine phosphosites in SARS-CoV-2 protein. **(C)** Phosphosites in nucleoprotein (N) of SARS-CoV-2. **(D)** Phosphosites in SARS-CoV-2 protein on serine (S), threonine (T), and tyrosine (Y) on n protein.

Production of virus proteins and their post-translation modification occurred in the host cell by utilizing the host cell enzyme (11). Stukalov et al. reported phosphorylation of known recognition motifs in 5 proteins of SARS-CoV-2, namely, M, N, S, NSP3, and ORF9b. Knowledge of post-translational modifications may help in finding antiviral therapies against SARS-CoV-2 (5). Davidson et al. reported first time phosphorylation in SARS-CoV-2 membrane-bound proteins such as M, nsp3, and S. Most of the phosphosites observed belong to serine phosphosite, followed by threonine phosphosites and single tyrosine phosphosites (**Figures 6A, B**).

### 3.1 N Proteins

Thirty-two phosphosites were detected on N protein, namely, 24 on serine and 8 on threonine phosphorylation sites (**Figures 6C, D**). N proteins of SARS-CoV-2 recruit GSK3 that is potentially related to phosphorylation of viral proteins. Additionally, ubiquitination was detected at K338 and phosphorylation was detected on S310 and S311 of SARS-CoV-2 N (5). Phosphorylation of N protein by GSK3B at serine-arginine (S-R)-rich motifs involves replication of coronavirus (3, 11). Nucleoprotein N has a large number of phosphorylation sites, and further analysis of residue 47–173 of PDB: 6vyo has suggested a small surface region about specific regulation and interaction changes (2). MAPKAPK2 and CAMK4 phosphorylate the sites on N proteins. Phosphosites T76, S78, S79, S105, T141, T166, S176, S180, S183, S184, and S186 occur in the RNA-binding region (**Figure 6C**). Furthermore, S310 belongs to the dimerization region and S176 belongs to the beta strand.

### 3.2 Spike (S) Proteins

The Spike protein plays an important role in attachment (12). Thirteen phosphosites were detected on the Spike protein, namely, 9 serine, 3 threonine, and 1 tyrosine phosphorylation site (**Supplementary Figures 2A, B**). Notably, the phosphosite present on the Spike protein plays an important role in the assembly of the trimer. The phosphosites T29, S31, S349, T791, and S816 are located on the surface while phosphosite T240 occurred below the disordered loop that helps in the formation of the loop by the addition of the negative charge. The Y789 and T791 found at the subunit interface may play an important role in regulating trimer assembly (4). Notably, CAMK4 and MAPKAPK2 phosphorylate the sites on S protein. Phosphosites T29, T240, S637, S640, and Y789 belong to the beta strand (**Supplementary Figure 2A**). Phosphorylation sites S459 of SARS-CoV-2 belong to a receptor-binding motif that binds to human ACE2. Further phosphorylation sites S1196 belong to the coiled-coil region and Heptad repeat 2. Phosphorylation site S349 belongs to the RBD.

Notably, important phosphosites S816 belong to Fusion peptide 1. Additionally, R815-S816 is part of cleavage sites by the host and phosphorylation in S816 may alter the situation of cleavage for the host. SARS-CoV-2 has an insertion of 4 amino acids 680-SPRR-683 in the Spike protein that creates furin-like cleavage site 682-RRAR-685 (13–15). Notably, no phosphosite was detected at S680. Furthermore, removal of the furin cleavage site from the Spike protein was observed and may function as an important vaccine target and also play an important role in virus pathogenesis and zoonosis. There is a high probability to mutate in this region of Spike protein and generate more virulent variants (4). Cleavage of the SARS-CoV-2 Spike protein occurs before virus exits the cell because of the occurrence of a furin-like cleavage site at the S1/S2 boundary. Notably, the absence of the S1/S2 furin cleavage site in the SARS-CoV-2 Spike protein and other coronaviruses causes them to exit the cells with largely uncleaved Spike protein. Therefore, cleavage needs to occur before or during cell entry (4, 6, 16, 17).

### 3.3 NSP Proteins

Notably, nsp3 is important for replication and transcription machinery and it has five phosphorylation sites, namely, T504, S660, S661, S794, and S1826. Furthermore, one phosphosite was detected on NSP 14 at serine (S56). A phosphorylation site on serine (S5) in NSP9 is present in the interface region supported by the analysis of the homodimer structure (PDB: 6W4B) (18). One phosphosite (S5) was detected in NSP-6 (SARS-CoV-2 protein homolog) in the host cell while SARS-CoV-1 protein 6 was suggested to accelerate the infection in the murine model (19). Large polyprotein 1b has one phosphosite S5981 in NSP11 and two other phosphosites (S723 and S2644) with unknown function region. Notably, it would be nice to know the occurrences of phosphorylation either before cleavage or after the cleavage (2). Nsp12 is involved in the replication of the viral genome by encoding the RNA-dependent RNA polymerase (RdRp). During the infection, the majority of protein interactors of Nsp12 showed a decrease in phosphorylation. Several interacting proteins of Nsp12 are involved in RNA processing such as LARP4B and CRTC3 that may have a functional role (1).

### 3.4 Other Proteins: Membrane (M) Protein, 3a, and 9b

Seven phosphosites were detected on M protein, namely, 5 on serine and 2 on threonine phosphorylation sites (**Supplementary Figure 3A**). The membrane protein M has high activity modification surface as phosphorylation in nearby 3 serines at the C-terminal and cytoplasmic region. Furthermore, the M proteins play an important role in the formation of a viral particle. Four phosphosites were detected on ORF 9b protein, namely, 3 on serine and 1 on a threonine phosphorylation site (**Supplementary Figure 3B**). In SARS-CoV-2, two phosphosites were observed on Protein 9b while the function is not known (2). S50 and S53 belong to the nuclear export signal motif and beta strand. Additionally, S72 also belongs to the beta strand. Furthermore, 2 phosphosites were detected on ORF 3a protein including one each on serine and threonine (**Supplementary Figure 3C**). The transmembrane protein 3a was phosphorylated on the luminal side. Furthermore, 3 phosphosites were detected on serine including S723, S2644, and S5981 in replicase polyprotein 1ab (Rep) (**Supplementary Figure 3D**).

## 4 Phosphorylation Studies of SARS-CoV-2-Infected Host Cells

Bouhaddou et al. have reported 4,624 phosphosites in 3,036 phosphoproteins using the SARS-CoV-2-infected chimpanzee Vero E6 cell line (1). Furthermore, Klann et al. have reported 15,093 phosphosites in 7,150 phosphoproteins in human colon cancer Caco-2 cells. Notably, 16,715 phosphopeptides with a significant increase of 2,197 phosphopeptides and a decrease of 799 phosphopeptides upon infection were also reported. SARS-CoV-2 infections cause an increase of more than 2,000 phosphopeptides in humans; however, their protein levels remained constant with signaling events and differential modification activities reported for phosphoproteins (2).

Hekman et al. reported phosphoproteomics studies of SARS-CoV-2-infected [at the air–liquid interface (ALI)] iAT2s. They preferred studies on iAT2s as AT2s are tough to maintain in culture. Notably, the culture of iAT2s at ALI maintains properties of self-renewal and also retains the transcriptional program of AT2s (3). An interesting study by Hekman et al. found 8,471 quantified host human proteins with 8 SARS-CoV-2 proteins along with 14,289 phosphosites on 2,703 host human phosphoproteins and 2 viral phosphoproteins (3). Furthermore, Stukalov et al. reported 16,399 detected phosphosites including 4,643 significant changes in host human lung cancer A549 cells expressing ACE2 (A549-ACE2 cells after infection with SARS-CoV-2 or SARS-CoV) (5).

### 4.1 Host Human Kinases

Host human kinases are responsible for causing phosphorylation modifications of viral proteins. SARS-CoV-2 infection makes heavy and significant changes in phosphorylation of both host human and viral proteins. Therefore, targeting the relative host human kinases may lead to new therapeutic strategies (2). Furthermore, SARS-CoV-2 infection significantly increases phosphorylation in carbon metabolism (2). Bioinformatics analysis suggested that nucleoproteins may be modified by CMGC kinases including casein kinase II (CK2). Notably, CK2 kinase has been reported as an interaction partner of nucleoprotein when expressed in cells (10). This shows that inhibition of CK2 kinases may help to understand the functional interaction between the viral protein and human kinases (2). Furthermore, SARS-CoV-2 infection enriched these kinases in cells (5).

Interestingly, three major clusters were identified on the basis of phosphorylation and proteome studies. Endocytic pathway, ErbB1 (EGFR) signaling, platelet-derived growth factor receptor (PDGFR), vesicle trafficking, and metabolism were major signaling pathways of the first cluster. Alteration in signaling activities is responsible for increased phosphorylation but not due to differences in the abundance of proteins. The proteins that are decreased in phosphorylation are related to the second cluster including cell cycle and translation initiation. Splicing machinery is significantly reshaped after SARS-CoV-2 infection and related to the third cluster (20–24). Studies have found that pladienolide B inhibits splicing and decreases the SARS-CoV-2 pathogenic effects (2).

### 4.2 Regulatory Mechanism of SARS-CoV-2 Infection

Data collected on SARS-CoV-2 infection especially proteins, mRNA, phosphorylation, and ubiquitination suggested its regulatory mechanism. Proteins involved in MAPK pathways including SRC, MAP2K1, JUN, and MAPKAPK2; autophagy signaling including OPTN, DEPTOR, LAMTOR1, and RICTOR; and viral entry including ACE2 and RAB7A were highly regulated. Notably, SARS-CoV-2 infection causes an increase in phosphorylation at S72 of RAB7A that helps in the endosomal trafficking of ACE2 to plasma membranes compared to SARS-CoV (25, 26). Additionally, SARS-CoV-2 infection causes phosphorylation at the important S33 residue of antiviral kinase EIF2AK2 (or PKR) that may be responsible for higher growth kinetics compared to SARS-CoV (27). Several phosphosites that are observed and known on central kinases including cyclin-dependent kinases, AKT, MAPKs, ATM, and CHEK1 are involved in various functions such as cell cycle, cell growth, motility, stress, and DNA damage response (5).

#### 4.2.1 MAPK Cascade

SARS-CoV-2 hijacked the host cellular pathway and activated host p38 MAPK cascade activity and closure of mitotic kinases. Furthermore, with the help of the budding virus, it energizes CK2-containing filopodial protrusions. The phospho analysis helps to identify potential antiviral compounds. Furthermore, pharmacologic inhibition had been observed for several kinases including p38, CK2, CDK, AXL, and PIKFYVE kinases (1). AXL regulates several signaling pathways including PI3K, Ras/ERK, and p38 (28, 29). The p38/MAPK activity has increased in severe COVID-19 along with the increase of IL-6, IL-10, and TNF-α with a decrease of lymphocyte or lymphopenia. Inhibitors of p38 MAPK, CK2, PIKFYVE, and CDKs showed significant antiviral efficacy. The p38/MAPK inhibition targets multiple unknown mechanisms during COVID-19 pathogenesis including suppression of cytokine production and impairment of viral replication (1).

#### 4.2.2 Cell Cycle Arrest

The majority of SARS-CoV-2 viral proteins were increased while host proteins were decreased in abundance after infection. Several observed phosphosites were regulated by kinases including casein kinase 2 (CK2), cyclin-dependent kinase (CDK), and protein kinase C (PKC), which may have an important role in regulating viral replication (1). SARS-CoV-2 infection causes a reduction in CDK1/2 activities that leads to S/G2 phase arrest that helps in viral replication and progeny production. The arrest of the S/G2 phase helps to provide enough supply of nucleotides and other essential host DNA repair and replication proteins (30). Furthermore, these viral properties are similar to other coronaviruses, bronchitis viruses, and other RNA viruses (1).

Bouhaddou et al. infected the Vero cells with SARS-CoV-2 for 24 h, and the amount of DNA was measured by DAPI staining and flow cytometry. Furthermore, the cell increase in the S phase, at G2/M transition, and the decrease in cells in the G0/G1 phase have again confirmed S/G2 phase arrest of the cell cycle (1). The abundance of major viral proteins was significantly increased while the majority of the host proteins were decreased, suggesting inhibition of host mRNA translation as other viral infections (31, 32). The downregulation of host proteins such as APOH, CD9, TSPAN14, AHSG, SERPINA1, and A2M was involved in the regulation of platelet, thrombosis, and anti-coagulation (33–35). Therefore, COVID-19 causes a higher risk of blood coagulation and stroke (36).

#### 4.2.3 Growth Factor Receptor Signaling

Klann et al. (2020) reported that SARS-CoV-2 proteins are highly phosphorylated in host cells. Furthermore, SARS-CoV-2 infection initiates the growth factor receptor (GFR) signaling and downstream pathway. Inhibitors of GFR downstream signaling cause disruption of SARS-CoV-2 replication in cells. Notably, host signaling plays an important role in the disturbance of virus replications (2). The SARS-CoV-2 infection was carried out for 24 h in Caco-2 cells and used iron-loaded nitrilotriacetic acid (Fe-NTA) for the enrichment of phosphopeptide (2). Notably, GFR signaling was highly phosphorylated while the mechanism to regulate SARS-CoV-2 infection by GFR signaling is not known. EGFR and its downstream signaling protein can be targeted by 28 clinically approved drugs that are mostly used in cancer therapy. GFR signaling activates several other signaling pathways such as RAF/MEK/ERK MAPK, phosphatidylinositol 3-kinase (PI3K), protein kinase B, and mTORC1 signaling to regulate proliferation (**Figure 3**) (2).

#### 4.2.4 TGF-β, Interferon, and EGFR

Stukalov et al. have reported phosphosites on five SARS-CoV-2 proteins including M, N, S, NSP3, and ORF9b (5). SARS-CoV-2 infection of an A549-ACE2 cell causes significant expression of FN1 and SERPINE1 that may be related to recruitment of TGF-β factors. Furthermore, it suggests that SARS-CoV-2 regulates and is linked to TGF-β signaling (37, 38).. The ORF8 of SARS-CoV-2 dysregulated TGF-β pathway that is involved in tissue fibrosis and ORF3 of SARS-CoV-2 dysregulated autophagy. Furthermore, attempts were made to identify the potential drug candidates among kinase and metalloprotease inhibitors (5).

ORF3, ORF6, ORF7a, ORF7b, and ORF9b play a significant role in the inhibition of interferon (IFN-α and IFN-β) induction or signaling that disrupts the antiviral immunity. ORF9b causes mitochondrial dysregulation (2, 39–41). Regulation of TGF-β and EGFR pathways modulates important activities including motility, cell survival, and innate immune responses after virus infection. Network analysis suggested the link between ORF8 and ORF3 that binds to TGF-β-associated factors (including TGF-β1, TGF-β2, LTBP1, TGFBR2, FURIN, and BAMBI), distinctive expression of extracellular matrix regulators (including FERMT2 and CDH1), and increase of fibrinogens (both FGA and FGB), fibronectin (FN1), and SERPINE1 (5, 42).

Regulation of TGF-β and EGFR pathways was further supported by the increase in protein phosphorylation related to MAPK signaling such as SHC1, SOS1, JUN, MAPKAPK2, and p38, and to receptor tyrosine kinase signaling such as PI3K complex members PDPK1 and RPS6KA1, and the increase in expression of JUN, FOS, and EGR1. Phosphorylation on serine residue was observed in SHC1 (S139), SOS1 (S1134/S1229), JUN (S63/S73), PDPK1 (S241), and RPS6KA1 (S380), while phosphorylation on threonine residues was observed in MAPKAPK2 (T334) and p38 (T180/Y182) (**Figure 3**). The integrin signaling and activation of YAP-dependent transcription affect TGF-β and EGFR signaling in a time-dependent manner with SARS-CoV-2 infection. Furthermore, it plays a role in virus replication and fibrosis caused by COVID-19 (5, 38, 43).

#### 4.2.5 Autophagy

The NSP6 plays an important role in SARS-CoV-2-induced autophagy (44). The ORF3 protein inhibits autophagic flux of SARS-CoV-2 causing accumulation of autophagy receptors including SQSTM1, GABARAPL2, and NBR1. Furthermore, it is very similar to the accumulation of MAP1LC3B in SARS-CoV-2-infected cells. An ORF3 protein interacts with the HOPS complex for autophagosome–lysosome fusion, differential phosphorylation of important regulatory sites including TSC2 and mTORC1 complex, and ubiquitination of important components including MAP1LC3A and GABARAPL2. Furthermore, it plays a valuable role in protein degradation. The risk of arterial thrombosis is increased by the accumulation of APOB levels that contribute to the failure of vital organs including lung, heart, and kidney in COVID-19 patients (45–47).

#### 4.2.6 Interaction Network of the Major Proteins Including Phosphoprotein Involved in COVID-19

We have used STRING (48) at a high confidence score (≥70%) cutoff to know about the protein–protein interactions (**Figure 4**, **Supplementary Table 1**) among the major proteins including phosphoproteins involved in COVID-19. **Figure 4** shows that the proteins MMP2, A2M, ACTR2, AHSG, AKT1, APOB, APOH, ATM, ATR, AXL, BAMBI, CDH1, CDK1, CDK2, CDK3, CDK5, CDK7, CHEK1, CSNK1A1L, CSNK2A1, DEPTOR, EGFR, FERMT2, FGA, FGB, FLT3, FN1, FOS, FURIN, GABARAPL2, GSK3B, HRAS, IL10, IL6, JUN, LAMTOR1, LTBP1, MAP1LC3A, MAP1LC3B, MAP2K1, MAP2K2, MAP2K7, MAPK1, MAPK14, MAPKAPK2, MMP2, MMP9, MTOR, NBR1, OPTN, PDPK1, PIK3CA, PIKFYVE, PRKCA, PTK2B, RAF1, RICTOR, RPS6KA1, SERPINA1, SHC1, SOS1, SQSTM1, SRC, TGFB1, TGFB2, TGFBR2, TNF, TSC2, and TSPAN14 form protein–protein interaction networks at high confidence interaction scores (≥70%).

## 5 Interplay of Phosphorylation and Ubiquitination

The interplay of phosphorylation and ubiquitination was observed in host human proteins. SARS-CoV-2 infection causes ubiquitination on 6 lysine residues after 24 h of infection along with phosphorylation of 2 serine residues (S695 and S991) and 1 threonine residue T693 at 24 and 36 h of infection. Vimentin has exhibited pronounced phosphorylation and ubiquitination patterns on various residues on early (S420) or late (S56, S72, and K334) stages of infection and has importance in the entry of coronavirus and pathogenesis (5, 49–51). It was found that 21 SARS-CoV-2 proteins were ubiquitinated including N, S, NSP2, and NSP3. Interestingly, the possibility of cross-talk between ubiquitination and viral protein functions was observed. Interaction between SARS-CoV-2 and host human E3 ligase proteins was observed such as SARS-CoV-2 ORF3 and TRIM47, WWP1, WWP2, and STUB, and SARS-CoV-2 M and TRIM7. Furthermore, it was observed in deubiquitinating enzymes such as SARS-CoV-2 ORF3 and USP8, SARS-CoV-2 ORF7a and USP34, and SARS-CoV N and USP9X. Significant ubiquitination was observed on S proteins including K97, K528, K825, K835, K921, and K947 that belong to functional domains including the N-terminal domain, C-terminal domain (CTD), fusion peptide, and heptad repeat 1 domain (5).

## 6 Kinase Inhibitor as Potential Therapeutics for SARS-CoV-2

SARS-CoV-2 infection causes phosphorylation and regulates several important signaling pathways of the host. Therefore, achieving the result by targeting the downstream signaling pathway has more benefit as it does not disturb other signaling pathway functions. Several enzymes can be used as a target for COVID-19 therapeutics including PI3K¸ mTOR, growth factor receptor, RAF, MAP2K2, FLT3, AXL, AKT, and matrix metalloprotease MMP2 and MMP9. Some inhibitors target viral replications and cytopathic effects during infection.

Pictilisib, an inhibitor of PI3K, and omipalisib, an inhibitor of PI3K and mTOR, inhibited viral replication. Sorafenib inhibits growth factor receptors and RAF, while RO5126766 inhibits both mitogen-activated protein kinase (MAP2K2 or MEK) and the RAF. Sorafenib and RO5126766 both inhibited viral replications and cytopathic effects during infection (2).

Specific inhibitors for various kinases showed that antiviral activities are under various stages of a clinical trial such as silmitasertib inhibitor of CK2; FDA-approved gilteritinib and bencentinib inhibitors of AXL; ARRY-797, MAPK13-IN-1, SB203580, and ralimetinib inhibitors of p38; apilimod inhibitor of PIKFYVE; and dinaciclib inhibitor of CDK (2). Furthermore, these drugs earlier made for different diseases are now repurposed for COVID-19 including silmitasertib for several cancers, ARRY-797 for cardiomyopathy, and gilteritinib for treatment of acute myeloid leukemia.

Notably, gilteritinib, ipatasertib, prinomastat, and marimastat have shown significant antiviral activity by inhibition of SARS-CoV-2 replication without any minor effect on cell growth. Gilteritinib is an inhibitor of FLT3 and AXL, while ipatasertib is an AKT inhibitor. Gilteritinib and tirapazamine showed antiviral activities against both SARS-CoV and SARS-CoV-2 (5). Prinomastat is a specific inhibitor of MMP2 and marimastat is a specific inhibitor of MMP9. Interestingly, both these matrix metalloprotease (MMP) inhibitors selectively showed significant antiviral activity against SARS-CoV-2 but no viral activity against SARS-CoV. Furthermore, MMP activities are associated with characteristics of COVID-19 including TGF-β activation, alveolar damage, pleural effusions, and neuroinflammation (5, 52–54).

The STRING (48) plot (**Figure 5**, **Supplementary Table 2**) provides protein–protein interactions among the therapeutic targets for COVID-19 with the cutoff of high confidence scores (≥70%). **Figure 5** shows that the proteins FLT3, AXL, MAP2K2, PIKFYVE, AKT1, MAPK14, MTOR, MAP2K7, EGFR, MMP9, MMP2, and TGFB1 form protein–protein interaction networks at high confidence interaction scores (≥ 70%).

## 7 Conclusion

The COVID-19 pandemic has been ongoing for years and we are still looking for better therapeutics. SARS-CoV-2 causes several changes inside the cell including heavy phosphorylation in the protein of the host cell. Furthermore, about 70 phosphorylation sites were detected in the majority of viral proteins especially in N and S proteins. More than fifteen thousand phosphorylation sites were detected in multiple studies belonging to several thousands of host phosphoproteins and multiple interconnected signaling pathways. A systematic study of the dynamic change in phosphorylation site and phosphoproteins will help to find suitable therapeutics of COVID-19, especially suitable kinase inhibitors. SARS-CoV-2 affects various kinases including CMGC, CK2, CDK, and PKC, and also regulates important signaling pathways including MAPK cascade, GFR signaling, TGF-β, AKT, Interferon, EGFR, and Autophagy.

Furthermore, alterations in signaling activities are responsible for hyperkinase activation and much higher phosphorylation. Notably, host human kinase also phosphorylates virus proteins. SARS-CoV-2 utilizes host cell enzymes for production of significant high viral protein and their phosphorylation. Specific inhibitors of upregulated kinases and interconnected signaling pathways can be potential therapeutics of COVID-19 such as pictilisib (inhibitor of PI3K), omipalisib (inhibitor of PI3K and mTOR), sorafenib (inhibits growth factor receptor and RAF), RO5126766 [inhibits both mitogen-activated protein kinase (MAP2K2 or MEK) and the RAF], gilteritinib (inhibitor of FLT3 and AXL), ipatasertib (AKT inhibitor), prinomastat (specific inhibitor of MMP2), and marimastat (specific inhibitor of MMP9). These inhibitors either alone or in a combinatorial approach will behave as potential therapeutics for the ongoing COVID-19 pandemic.

## 8 Research Design and Methods

The major proteins involved in COVID-19 along with the therapeutic targets for COVID-19 were searched using PubMed.gov. The interaction network involving major proteins involved in COVID-19 along with the therapeutic targets for COVID-19 was generated using the STRING database (48) (https://string-db.org/) in order to obtain protein–protein interaction information. This information was based on text mining, co-occurrence, co-expression, experiments, gene fusion, neighborhood, and databases with a cutoff at high confidence interaction scores (≥70%).

## Author Contributions

BC and ST have conceived the idea and wrote the manuscript. All authors contributed to the article and approved the submitted version.

## Conflict of Interest

The authors declare that the research was conducted in the absence of any commercial or financial relationships that could be construed as a potential conflict of interest.

## Publisher’s Note

All claims expressed in this article are solely those of the authors and do not necessarily represent those of their affiliated organizations, or those of the publisher, the editors and the reviewers. Any product that may be evaluated in this article, or claim that may be made by its manufacturer, is not guaranteed or endorsed by the publisher.

## Supplementary Material

Supplementary Figure 1
**(A)** Phosphosites detected in SARS-CoV-2 infected host cell line Protein. **(B)** Phosphosites and **(C)** Phosphoproteins detected in SARS-CoV-2 Proteins.

Supplementary Figure 2
**(A)** Phosphosites Spike glycoprotein (S) of SARS-CoV-2, **(B)** Phosphosites in SARS-CoV-2 Protein on Serine (S), Threonine (T) and Tyrosine (Y) on Spike protein of SARS-CoV-2.

Supplementary Figure 3 Phosphosites in **(A)** Membrane protein (M), **(B)** ORF9b protein (9b), **(C)** ORF3a protein (3a), **(D)** Replicase polyprotein 1ab (Rep) of SARS-CoV-2.

Supplementary Table 1 Details of Interaction network of major proteins involved in COVID-19 at high confidence scores.

Supplementary Table 2 Details of Interaction network of therapeutic targets for COVID-19 at high confidence scores.
