# Supplementary figures and images for: SARS-CoV-2 Infection Triggers Phosphorylation: Potential Target for Anti-COVID-19 Therapeutics
Source: Front Immunol. 2022 Feb 17;13:829474. doi: 10.3389/fimmu.2022.829474 (PMC8891488; doi:10.3389/fimmu.2022.829474)

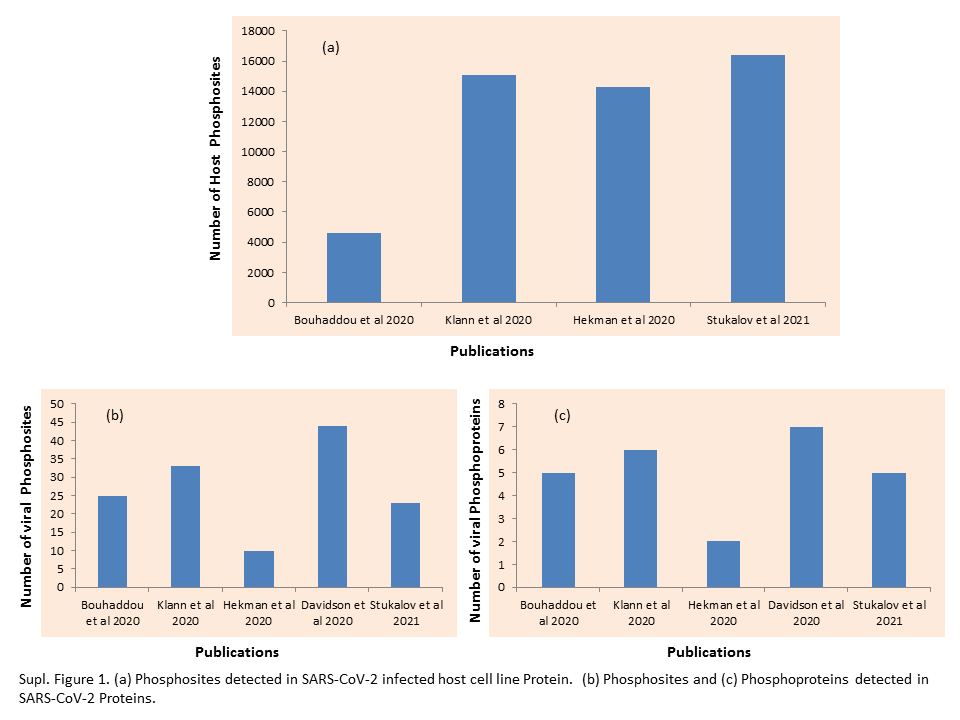

Supplement: Supplementary Figure 1 — (A) Phosphosites detected in SARS-CoV-2 infected host cell line Protein. (B) Phosphosites and (C) Phosphoproteins detected in SARS-CoV-2 Proteins. [file Image_1.tif]

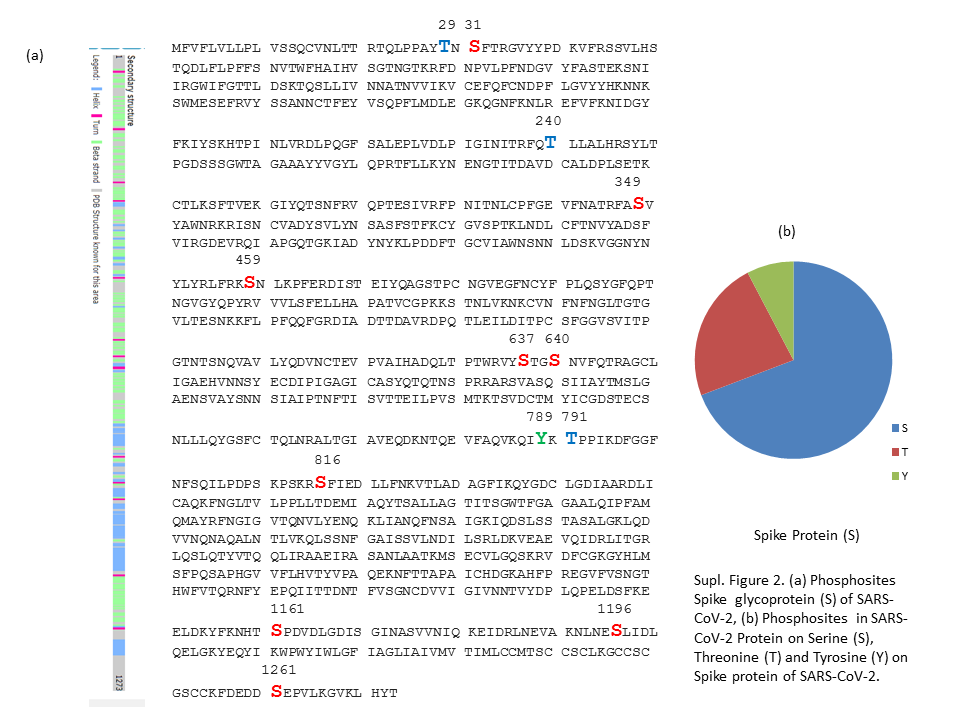

Supplement: Supplementary Figure 2 — (A) Phosphosites Spike glycoprotein (S) of SARS-CoV-2, (B) Phosphosites in SARS-CoV-2 Protein on Serine (S), Threonine (T) and Tyrosine (Y) on Spike protein of SARS-CoV-2. [file Image_2.tif]

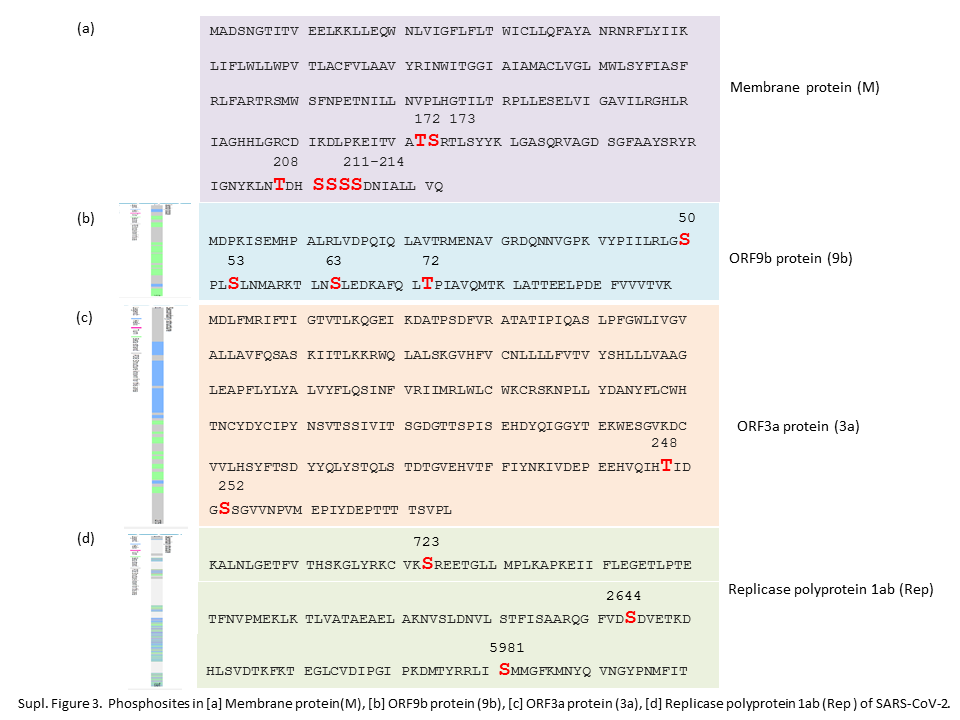

Supplement: Supplementary Figure 3 — Phosphosites in (A) Membrane protein (M), (B) ORF9b protein (9b), (C) ORF3a protein (3a), (D) Replicase polyprotein 1ab (Rep) of SARS-CoV-2. [file Image_3.tif]
